# Reply to Eltawil et al. Comment on “Iacobescu et al. Evaluating Binary Classifiers for Cardiovascular Disease Prediction: Enhancing Early Diagnostic Capabilities. *J. Cardiovasc. Dev. Dis.* 2024, *11*, 396”

**DOI:** 10.3390/jcdd13010047

**Published:** 2026-01-15

**Authors:** Paul Iacobescu, Virginia Marina, Catalin Anghel, Aurelian-Dumitrache Anghele

**Affiliations:** 1 Doctoral School of “Dunărea de Jos” University of Galati, 800201 Galati, Romania; anghele_aurelian@yahoo.com; 2 Medical Department of Occupational Health, Faculty of Medicine and Pharmacy, “Dunărea de Jos” University, 800201 Galati, Romania; virginia.marina@ugal.ro; 3 Department of Computer Science and Information Technology, “Dunărea de Jos” University of Galati, 800201 Galati, Romania

We appreciate the careful and critical reading of our work by Eltawil et al. and the opportunity their commentary provides to clarify, refine, and further substantiate the methodological underpinnings of our study [1,2]. At the same time, in order to preserve a balanced scientific record, it is necessary to distinguish between (i) the valid and useful points raised in their critique and (ii) those extrapolations which, in our view, go beyond what the empirical evidence and the theoretical properties of the methods can support. In this letter, we openly acknowledge where our original presentation was ambiguous and where numerical results were affected, while providing a detailed, point-by-point clarification that addresses and, where appropriate, corrects claims in the commentary that are not justified by the reconstructed data or by the underlying machine learning theory.

First, regarding the most serious point—data leakage through improper resampling—we acknowledge that the wording of our original methods section allowed a reasonable reader to infer that SMOTE-ENN had been applied at the level of the full dataset prior to the 70:30 train–test split. The sentence “Following data cleaning, feature engineering, and addressing class imbalance, the next step was data transformation… Finally, the dataset was divided into training and testing subsets (70:30)” was intended as a high-level description of the workflow, but we recognize that, as written, it is compatible with a pre-split oversampling scheme. The commentary correctly notes that, if SMOTE-ENN were indeed applied globally before splitting, this would represent a violation of the principle that any operation “learning” from the data (including class-imbalance correction) must be confined strictly to the training portion.

We consider it essential not to minimize this concern. In response, we have reconstructed the entire pipeline with a strict “split-first” design. In the revised workflow, the BRFSS 2021 data are first cleaned and partitioned into training and test subsets (70:30). All subsequent operations that can induce data-dependent transformations—including SMOTE-ENN, feature scaling, and hyperparameter tuning—are restricted to the training set only. For models tuned via cross-validation, SMOTE-ENN and scalers are fitted anew within each training fold and applied only to the corresponding internal validation fold; the held-out external test set remains completely untouched by any resampling or scaling procedure. This revised pipeline is fully aligned with the guidelines also emphasized by the commentators: split early, apply oversampling only on training data, and keep the test set sacrosanct as a true proxy for unseen data.

Starting from the original BRFSS 2021 dataset, we re-estimated all models with the same hyperparameters, architectures, and threshold optimization procedures as in the original study, first performing a 70/30 train–test split and then applying the SMOTE-ENN algorithm exclusively within the training data (and within the training folds during cross-validation). Table 1 summarizes the updated performance of all classifiers on the 30% held-out test set, which retains the original prevalence of cardiovascular disease and is never exposed to any resampling or scaling step.

This revised table is important for evaluating the commentary’s conclusions. Once the pipeline is strictly train-only for SMOTE-ENN and all other data-dependent transformations, the performance of every classifier falls into a range widely recognized as realistic for cardiovascular risk prediction on BRFSS and similar population health surveys. Nothing in these numbers suggests catastrophic leakage or an “artificially easy” classification problem. Instead, we see exactly what one would expect for a challenging, noisy, imbalanced, multifactorial outcome: ensemble methods (XGBoost, Random Forest) at the top, a reasonably well-performing ANN, followed by linear and naive probabilistic models, and a kNN classifier exhibiting moderate performance governed by local geometry.

In this sense, we fully agree with the commentators on one key point: the near-perfect kNN results originally reported should not be retained as valid. Those extreme values were an artefact of the original pipeline and must be superseded by the more moderate, corrected estimates provided above. We consider it important to state this openly. Where we depart from the commentary, however, is in their implication that this correction empties the study of scientific content. The reconstructed metrics show that when the evaluation protocol is properly enforced, the comparative structure of the models remains stable and informative, and the main substantive conclusions of the paper—namely, the relative strengths and weaknesses of different classifier families for BRFSS-based CVD prediction—still hold.

A second pillar of the commentary is the argument that the configuration k = 2 for the kNN classifier is “inherently suspect” and, by itself, almost diagnostic of leakage. We respectfully but firmly disagree with this inference. The kNN algorithm does not impose any theoretical lower bound on k beyond k ≥ 1. The appropriate value of k is not a monotonic function of sample size; rather, it is determined by the interplay of several factors: the intrinsic dimensionality of the data, the fragmentation and density of the minority-class manifold, the degree of local heterogeneity in the feature space, and the trade-off between bias and variance along the decision boundary. In large, heterogeneous biomedical datasets such as BRFSS, it is entirely plausible that very small neighborhoods capture clinically meaningful micro-patterns—especially in the presence of complex interactions between age, comorbidities, lifestyle and socioeconomic variables.

What matters, therefore, is not the absolute magnitude of k, but the behavior of the model under that configuration. If k = 2 were being spuriously favored because synthetic points had effectively duplicated test instances, we would expect to see kNN with k = 2 achieving implausibly high metrics in the corrected pipeline as well—metrics approaching those originally reported. That is not the case. Under the fully corrected workflow, kNN with k = 2 yields an ROC-AUC of 0.714, accuracy of 0.80, and an F1-score of 0.27, with precision and recall exhibiting the usual tension in imbalanced settings. These numbers are not even remotely in the “too-good-to-be-true” regime; they are modest, consistent with independent analyses, and entirely in line with the known difficulty of the task.

This is a critical empirical counterargument: k = 2 appears in the final table, yet the evaluated performance is moderate, not extreme. That combination is incompatible with the narrative that k = 2 serves as an automatic indicator of leakage. In reality, k = 2 simply reflects the fact that, under our feature representation and in the presence of SMOTE-ENN applied correctly to the training folds, the most informative neighborhoods for this particular problem are very local. Some datasets support larger k; ours does not have any obligation to do so, and the corrected metrics offer no evidence that k = 2 is pathological.

The commentary further asserts that SMOTE-ENN, when mis-sequenced, “creates synthetic points nearly identical to test samples” and that this, together with k = 2, explains the original, unusually high performance. Here, we believe the authors overstate what can be inferred from the properties of the algorithm. SMOTE generates synthetic samples along line segments between minority-class neighbors in feature space. It does not extrapolate outside the convex hull of observed minority points and certainly has no access to the test labels or features during training when used correctly. ENN deletes noisy or borderline points based on nearest-neighbor disagreement; it does not generate new ones. For a synthetic point to be “nearly identical” to a test sample, that test point must already lie near the interior of the minority manifold, and the synthetic point must fall almost exactly at that location. While such coincidences are not impossible in principle, they are not systematically enforced by the algorithm and, more importantly, do not give rise to the type of dramatic performance inflation that would be required to support the stronger claims in the commentary under a corrected pipeline.

The fact that, once corrected, all models—including kNN—converge to realistic performance regimes indicates clearly that what we are dealing with is not a complete breakdown of methodological safeguards, but an over-interpretation of a single flawed configuration. When the model is re-evaluated under a pipeline that respects the train-only constraint for resampling, the BRFSS problem presents itself exactly as the literature would suggest: difficult, structured, and sensitive to model capacity and inductive bias.

A third theme of the commentary relates to our use of mean squared error (MSE) as the loss function for the ANN. The authors correctly note that binary cross-entropy (BCE) has become the dominant choice for binary classification tasks in modern deep-learning frameworks. We do not dispute this. However, the implication that the use of MSE reveals a conceptual misunderstanding, or that it meaningfully undermines the reliability of the ANN results, is, in our view, not supported by theory or by the empirical evidence we observe.

Historically, MSE has been one of the principal loss functions used to train multilayer perceptrons, including in classification contexts. Before probabilistic interpretations of the output layer became standard, many neural network models were trained as function approximators minimizing squared error between predicted scores and target encodings (e.g., {0, 1}). From a functional analysis perspective, MSE remains a well-behaved objective: it is continuous, differentiable, and its gradient promotes convergence toward a mapping that approximates the conditional expectation of the target given the input. In a binary setting, if the output layer is interpreted as a score in [0, 1], MSE minimization can still drive the network toward a useful discrimination function—even if BCE may be more natural from a probabilistic standpoint.

In the specific context of BRFSS-based cardiovascular prediction, several practical considerations made MSE a reasonable and deliberate choice in our original implementation. The dataset combines continuous, ordinal, and categorical features transformed through different encodings, resulting in a heterogeneous feature space. The output to be learned is not a finely calibrated probability estimate intended for direct risk communication to individual patients, but rather a discriminative score used primarily for comparing different model families within the same pipeline. Under such conditions, the primary requirements for the loss function are stability, monotonicity with respect to classification errors, and support for smooth gradient updates. MSE satisfies all of these.

Furthermore, we empirically verified that using MSE did not distort the relative placement of the ANN in the model hierarchy. When we experimentally replaced MSE with BCE in the corrected pipeline—keeping all other elements constant—the ANN’s ROC-AUC and F1-score remained essentially unchanged and its position relative to XGBoost, Random Forest, and Logistic Regression was preserved. This indicates that the choice of MSE did not drive any of the comparative conclusions. In other words, even if BCE is now more conventional and we agree that it is preferable for future implementations, MSE in our setting was not a methodological error; it was a defensible modeling choice whose impact is empirically negligible with respect to the broader findings.

We therefore maintain, respectfully but clearly, that the commentary’s attempt to treat the use of MSE as another major defect is overstated. It is entirely legitimate to prefer BCE and to encourage the community to adopt it as a standard; we are sympathetic to that view and we have adjusted our own configuration accordingly. But it does not follow that the ANN results reported under MSE are invalid or that they signal a fundamental misunderstanding of classification theory. The alignment of ANN performance under both losses contradicts that inference.

The final point raised by the commentary concerns the distinction between “validation” and “testing”. Here, we agree fully with the underlying principle: terminological precision matters. In the revised description, we now explicitly state that all hyperparameter tuning and model selection occur exclusively through k-fold cross-validation on the training set, and that the test set is reserved solely for a single, final evaluation. The internal “validation” referred to in the original text is now clearly corrected as “cross-validation within the training set,” preventing confusion. This adjustment improves the reproducibility of our work and brings our wording fully in line with current best practices.

Putting all these elements together, a more balanced picture emerges. The commentary correctly identifies one genuine problem: the ambiguity in our pipeline description, which, when implemented in the least favorable plausible way, can generate inflated performance for kNN. We have corrected that problem by adopting a strictly split-first pipeline, re-training all models, and reporting new test-set results that are realistic and consistent with external benchmarks. At the same time, several of the commentary’s stronger claims—such as treating k = 2 as proof of leakage, or characterizing the use of MSE as inherently erroneous—do not withstand scrutiny once the corrected metrics and the theoretical properties of the methods are taken into account.

Our revised results show that the underlying scientific question addressed by the paper remains sound. When properly evaluated, the ensembles perform best, the ANN is a competitive alternative, simpler models trail behind as expected, and kNN occupies a moderate but meaningful position. Rather than invalidating the study, the critique and our subsequent re-analysis have strengthened its methodological foundation and made explicit a number of modeling decisions that were previously implicit. We view this as a positive outcome: the commentary has helped refine the study, but it does not justify dismissing the main conclusions.

## Figures and Tables

**Table 1:** Performance of the re-evaluated classifiers on the 30% held-out BRFSS 2021 test set under a leakage-free pipeline. Best_threshold denotes the probability cutoff used to convert predicted probabilities to class labels, chosen on the resampled training set by maximizing the F1-score of the positive class.

Model	Best_Threshold	Accuracy	F1-Score	Precision	Recall	ROC-AUC
XGBoost	0.45	0.85	0.34	0.26	0.47	0.815
Random Forest	0.45	0.79	0.33	0.22	0.61	0.808
ANN	0.50	0.76	0.31	0.20	0.69	0.803
Logistic Regression	0.45	0.71	0.29	0.18	0.74	0.790
Naïve Bayes	0.45	0.66	0.26	0.16	0.77	0.770
kNN	0.55	0.80	0.27	0.20	0.46	0.714

## Data Availability

The manuscript has not been published elsewhere. Data are contained within the article.
